# An exploration of reformulation efforts by the food industry in Ireland

**DOI:** 10.1093/eurpub/ckac129.174

**Published:** 2022-10-25

**Authors:** D McMenemy, MR Sweeney

**Affiliations:** School of Nursing, Psychotherapy, and Community Health, Dublin City University, Donabate, Ireland

## Abstract

**Background:**

Previous research conducted in 2020 by our team examined the progress made by food manufacturers in reformulation between 2014 and 2017 (i.e., improving the nutrition profile of food staples by reducing salt, sugar, saturated fat and overall energy contribution). Our previous study showed improvements in salt and sugars levels across many food staples, but we found rising energy levels, total fat, and saturated fat in many food categories.

**Methods:**

This study aimed to explore the ongoing progress in reformulation between 2017 to 2021. We photographed the labels of food staples in supermarkets with the leading market share in the Republic of Ireland (Tesco's, Dunnes, SuperValu, Lidl, Centra, Aldi, and M&S). We extracted the data, collated it in an excel spreadsheet, and analysed it to examine the nutrients of interest to the study (i.e., salt, sugar, fat, saturated fat, energy, carbohydrates, protein, fibre, and micronutrients). We compared the levels captured at this time point with those previously recorded in 2017.

**Results:**

Eight hundred and seventy-two products were directly compared, including 80 spreads, 34 cereal snacks, 87 fruit juices, 193 cereals, 210 breads, 88 milks, and 169 yoghourts. This study shows that previously reported improvements in salt and sugar levels now appear to be going in the wrong direction.

**Conclusions:**

Fat and saturated fat levels that were once on the increase now appear to be reducing, possibly implying that as salt and sugar go up, fat levels go down and vice versa. This may relate to product taste and palatability.

**Key messages:**

• This study shows that previously reported improvements in salt and sugar levels now appear to be going in the wrong direction.

• Fat and saturated fat levels that were once on the increase now appear to be reducing, as salt and sugar go up, fat levels go down.

